# Transcriptome analysis of beta-lactamase genes in diarrheagenic *Escherichia coli*

**DOI:** 10.1038/s41598-019-40279-1

**Published:** 2019-03-06

**Authors:** Taru Singh, Praveen Kumar Singh, Shukla Das, Sayim Wani, Arshad Jawed, Sajad Ahmad Dar

**Affiliations:** 1 Department of Microbiology, University College of Medical Sciences (University of Delhi) & GTB Hospital, Delhi, India; 2 Department of Minimal Access and Bariatric Surgery, Fortis Flt. Rajan Dhall Hospital, New Delhi, India; 3 Research and Scientific Studies Unit, College of Nursing and Allied Health Sciences, Jazan University, Jazan, Saudi Arabia

## Abstract

Beta (β)-lactamases are the most important agents that confer drug resistance among gram-negative bacteria. Continuous mutations in β-lactamases make them remarkably diverse. We carried out the transcriptome analysis of 10 β-lactamase genes of Extended-Spectrum β-lactamases (ESBL), Metallo β-lactamases (MBL), and *AmpC* β-lactamases (ABL) in drug-resistant and sensitive diarrheagenic *E. coli* (DEC) isolates obtained from children up to 5 years of age. Out of the 10 β-lactamase genes, four belonged to ESBL (*TEM*, *SHV*, CTX, and *OXA*); three to MBL (*NDM*-1, *IMP*, and *VIM*); and three to ABL (*ACT*, *DHA* and *CMY*) class of genes. The different categories of DEC were estimated for β-lactamases production using a set of conventional phenotypic tests, followed by detection of their messenger RNA (mRNA) expression. The study revealed a direct correlation between mRNA expression of these genes and the presence of antibiotic resistance; also corroborated by mutation analysis of the *AmpC* promoter region. All the 10 β-lactamase genes showed a significant increase in their expression levels in resistant isolates, compared to those of the sensitive isolates, indicating their possible role in the disease pathogenesis. Increase in mRNA expression of β-lactamase genes, and thereby virulence, may be due to multifactorial parameters causing phenotypic as well as genotypic changes. Our study highlights the necessity of instantaneous detection of β-lactamase gene expression to curb the overwhelming threat posed by emergence of drug resistance amongst the commensal *E. coli* strains in children from developing countries for larger public health interest.

## Introduction

Widespread infections caused by antibiotic-resistant microbes are the biggest threats to public health in developing countries. The most widespread class of human antibacterial is the β-lactams. The development of resistance to β-lactam antibiotics in gram-negative pathogens, especially in *Escherichia coli* (*E. coli*), is a result of the production of β-lactamase enzymes, which endows the microbes with the ability to hydrolyze the β-lactam ring^1^. So, these β-lactamases are the real “Achilles heel” of antibiotic resistance in bacteria, killing thousands of people across the world every year^2^.

Extended-Spectrum β-lactamases (ESBLs) and plasmid-borne *AmpC* β-lactamases (ABLs) both can hydrolyze penicillin, cephalosporins, and bactams; whereas ABLs have a broader substrate profile, can degrade cephamycins^3^ and are resistant to β-lactamase inhibitors. Clavulanic acid (CA) inhibits ESBL but not ABLs^4^. The resulting β-lactam-resistant phenotype in *E. coli* is mainly a consequence of the acquisition of plasmid-mediated β-lactamases such as class A - ESBL (*TEM*, *SHV*, *CTX-M* and *OXA*), class C plasmid-mediated *AmpC* (*ACT*, *CMY* and *DHA*) or by the hyperproduction of the chromosomal *AmpC* enzyme, and class A, B or D carbapenemases^5,6^. Metallo β-lactamases (MBLs) - *VIM*, *IMP*, and *NDM*, reported from *Enterobacteriaceae* in the recent past, further limits the treatment options^7^.

In spite of the lower occurrence of the plasmid-mediated ABLs compared to ESBLs, they have been reported widely from different areas of the world. Carbapenemases are the β-lactamases that include MBLs and serine-β-lactamases (*KPC*, *OXA*, *GES*, etc.). The MBLs require zinc ion for their action, and these are inhibited by metal chelators like EDTA and thiol-based compounds but not by sulbactam, tazobactam, and clavulanic acid. Chromosomal or plasmid-mediated genes are responsible for MBL production, and these genes can be transferred to other gram-negative bacteria through horizontal gene transfer^8^.

The expression levels of β-lactamases in gram-negative bacteria are reported to be lower in comparison to gram-positive bacteria^9,10^. The presence of mutations in the promoter region is the most common mechanism responsible for hyperproduction of β-lactamases^11–14^. In clinical isolates, the overexpression of the chromosomal *AmpC* enzyme is primarily linked to *AmpC* promoter mutations^11,15,16^. In *E. coli*, the strength of the *AmpC* promoter generally defines the level of transcription of the *AmpC* gene^11,15^. The *E. coli* promoters harbor two hexamers (35 and 10 regions) of conserved sequences which play an essential role in gene transcription. The 35-consensus sequence is TTGACA, and the 10-consensus sequence is TATAAT; together they constitute the Pribnow box. The sequences that are closer to the consensus make stronger promoter. Overexpression of the *AmpC* gene can result in resistance to ampicillin, cefoxitin and expanded-spectrum cephalosporins. These mutations are not always redundant and sometimes can be misinterpreted or pass undetected.

The drug resistance genes have the potential to be used as important molecular markers for analyzing the prevalence and effect of the developed resistance. As reviewed elsewhere, the resistance mediated by β-lactamases is of particular concern because third-generation cephalosporins or higher generation antibiotics against β-lactams have long been used to treat *E. coli* infections successfully^17^. Further, there is a limited data available regarding β-lactamase genes in diarrheagenic *E. coli* (DEC) from children in developing countries, particularly India. All this demands effort to further understand the mechanisms involved in epidemiology of β-lactamase mediated resistance and specific resistant genotypes, both locally and globally. Due to limitations in phenotypic methods for perceiving antibiotic resistance, highly sensitive and efficient molecular methods are serving as the promising tools to detect bacterial mRNAs^18–21^. Gene expression in bacteria is complicated because of unstable nature and short half-lives of the bacterial mRNA as compared to eukaryotic mRNA^22^, limiting the detection rate, accuracy and approach of phenotypic detection to all species of gram-negative microorganisms^23,24^.

As data regarding expression of β-lactamase genes in *E. coli* isolates is scarce in children from India; we in this study intended to target 10 β-lactamase genes for expression analysis with an aim to predict a real-time scenario of the DEC response under antibiotic stress. We investigated the effect of antibiotic treatment on ESBL, MBL and ABL genes in resistant and sensitive isolates of DEC to analyze the differences in transcriptomes and also understand the survival of multidrug resistant bacteria. The findings will be useful in enabling clinicians to prepare specific strategies for surveillance and prevent development of drug resistance in children from developing countries, like India, for larger public health interest.

## Result

We in our previous study found that the most frequent category of DEC detected in paediatric population suffering from diarrhea was Enteropathogenic *E. coli* (EPEC) followed by Enteroaggregative *E. coli* (EAEC), Enterotoxigenic *E. coli* (ETEC) and Enterohemorrhagic *E. coli* (EHEC)^25^. Forty *E. coli* isolates each from children with diarrheal symptoms not receiving antibiotics (group 1); children receiving antibiotic therapy for 72 hours or more for reasons other than diarrhea (group 2); and healthy children (group 3) were studied for identification of the DEC isolates. The number of DEC isolates detected in each study group were 40, 39 and 27, respectively.

### Antibiotic susceptibility testing

Antibiotic susceptibility testing was performed on 16 antibiotic agents (Supplementary File - Table S1). It was found that highest resistance rate was observed in cefotaxime (55.83%) followed by gentamicin (25.83%), ampicillin (25%), norfloxacin (21.66%), amikacin (19.1%), piperacillin/tazobactam (17.5%) and imipenem (15%). Ceftazidime, ciprofloxacin, aztreonam and nalidixic acid showed similar antibiotic response (9% to 11.6%). In group 1 and 2, highest frequency of resistance was seen with cefotaxime in 67.5% and 82.5% isolates, respectively, while in group 3 it was highest with norfloxacin (25%).

### Quantitative reverse transcription Real-Time PCR (RT-qPCR) melting curve analysis

Individual analysis of the genes showed symmetrical peaks with some exception where frequently asymmetric peaks were observed. Each target gene, from DEC isolates, after PCR amplification presented with different melting curves and distinct peaks during melting analysis. The melting temperature (Tm) was as follows: *TEM*: 82.55 + 0.50; *SHV*: 86.26 + 0.65; *CTX*: 85.01 + 0.25; *OXA*: 80.15 + 0.62; *IMP*: 87.93 + 0.37; *NDM*-1: 78.01 + 0.56; *VIM*: 82.29 + 0.67; *ACT*: 82.35 + 0.58; *CMY*: 85.08 + 0.32; and *DHA*: 78.10 + 0.66 (Supplementary File - Figs S1–S3). The same Tm, for each gene, was detected with the positive control strains. Remarkably, little variation in Tm was observed among the different strains tested.

### Nucleotide accession numbers

Nucleotide sequences were compared against GenBank database by using Basic Local Alignment Search Tool (BLAST). Sequence of β-lactamase genes and reference genes were submitted to NCBI, and accession number were obtained for *TEM* (KX941097, KY753820); *CTX* (KY753817, KY883447, KY883448, KY883449, KY883450); *NDM*-1 (KY753818); *CMY* (KY753819); glyceraldehyde-3-phosphate dehydrogenase (*GAPDH*)-1 (KY775450); 16SrRNA (KY775448, KY775449, KY786039, KY786040, KY786041, KY786042, KY786043, KY786044, KY786045, KY786046, KY786047); *ACT* (KY883446); *SHV* (KY883445); and *OXA* (KY913604).

### Transcription analysis

**Figure 1:**
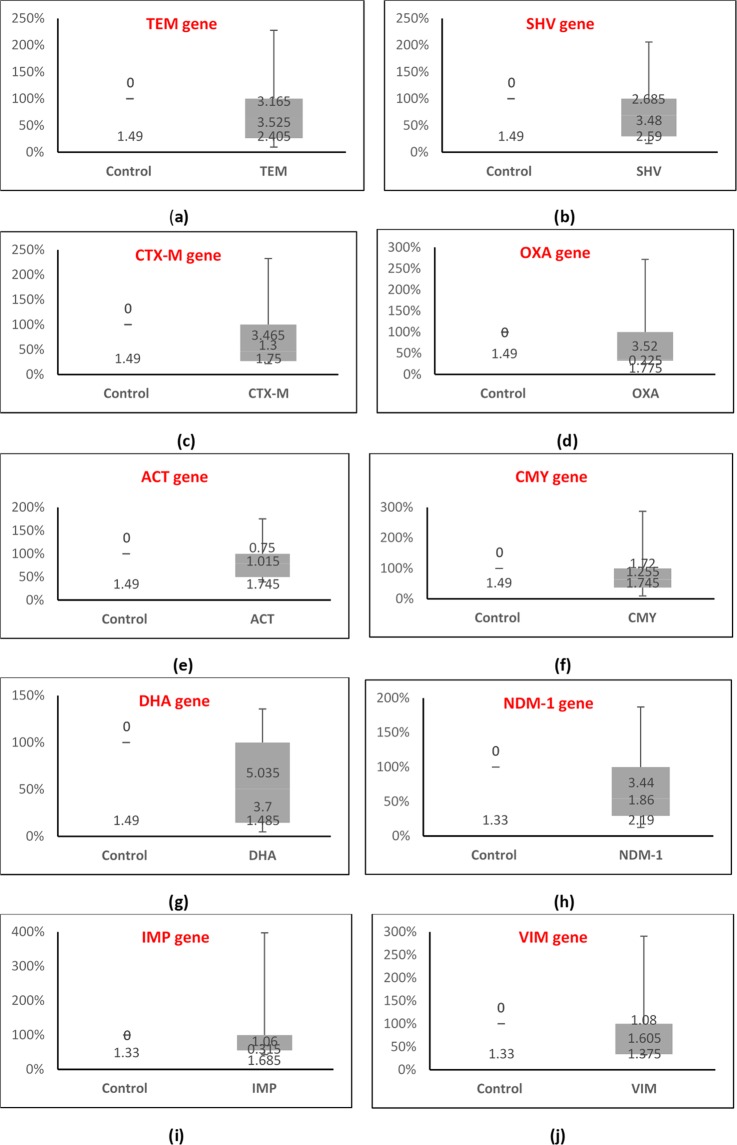
Relative expression of β-lactamase genes in drug-resistant DEC isolates as determined by qPCR and analyzed by the 2^−ddCt^ method. Box plots showing expression of (**a**) *TEM* gene, (**b**) *SHV* gene, (**c**) *CTX-M* gene, (**d**) *OXA* gene, (**e**) *ACT* gene, (**f**) *CMY* gene, (**g**) *DHA* gene, (**h**) *NDM*-1 gene, (**i**) *IMP* gene, and (**j**) *VIM* gene for 15 resistant DEC isolates.

**Figure 2:**
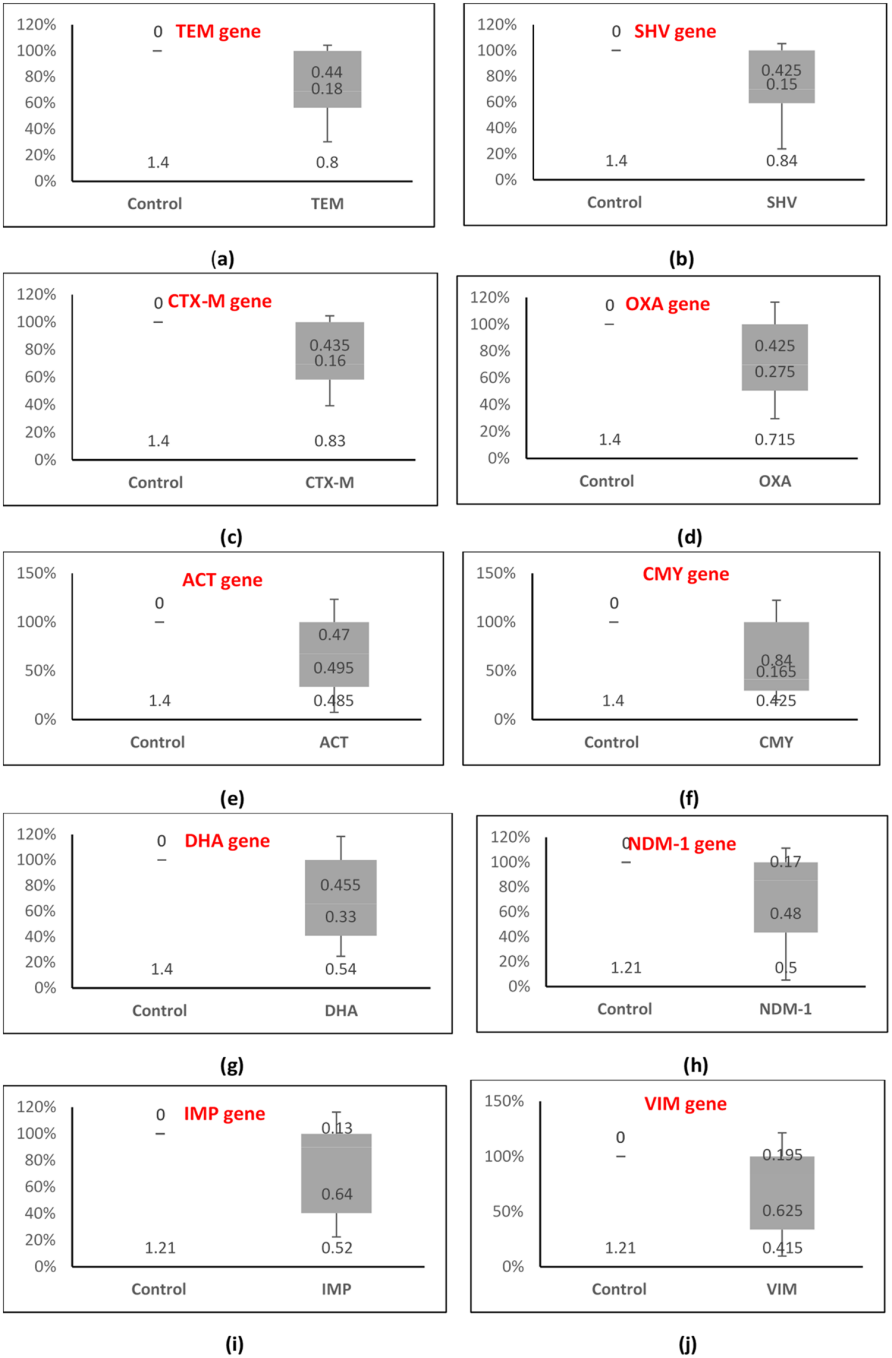
Relative expression of β-lactamase genes in drug-sensitive DEC isolates as determined by qPCR and analyzed by the 2^−ddCt^ method. Box plots showing expression of (**a**) *TEM* gene, (**b**) *SHV* gene, (**c**) *CTX-M* gene, (**d**) *OXA* gene, (**e**) *ACT* gene, (**f**) *CMY* gene, (**g**) *DHA* gene, (**h**) *NDM*-1 gene, (**i**) *IMP* gene, (**j**) *VIM* gene for 15 sensitive DEC isolates.

Fifteen DEC isolates from each group were studied for mRNA expression analysis. The relative expression of the related genes in 15 resistant isolates showed a high fold change (Fig. 1a–j). The level of expression in these isolates for *TEM* gene showed a range of 0.87–20.73; for *SHV* 1.43–18.03; for *CTX-M* 1.42–14.99; for *OXA* 1.41–14.99; for *ACT* 1.39–6.15; for *CMY* 0.44–13.56; for *DHA* 0.48–13.87; for *NDM*-1 0.92–14.00; for *IMP* 1.32–12.16; and for *VIM* 1.33–11.80-fold. The relative expression levels of these genes in sensitive isolates (Fig. 2a–j) showed a range of 0.43 to 1.48 for *TEM* gene; 0.34 to 1.49 for *SHV*; 0.56 to 1.49 for *CTX-M*; 0.42 to 1.65 for *OXA*; 0.11 to 1.59 for *ACT*; 0.29 to 1.75 for *CMY*; 0.33 to 1.57 for *DHA*; 0.23 to 1.28 for *NDM*-1; 0.29 to 1.50 for *IMP*; and 0.12 to 1.50 for *VIM* (Table 1). Average fold change in relative expression for each β-lactamase drug resistant and sensitive gene is shown in Supplementary File (Fig. S4). Both the internal control genes [16SrRNA and GAPDH] showed the stable level of expression at all the conditions. The mRNA transcripts for 16SrRNA and GAPDH genes gave similar signals for all isolates; however, different signals were obtained for different target genes. Overall, the mRNA expression of the target genes was found increased in the resistant isolates.

**Table 1:** Details of the β-lactamase genes studied along with the number of resistant and sensitive isolates with N-fold <1 (decreased expression), N-fold ranging from 1 to 1.99 (comparable expression) and N-fold ≥2 (increased expression).

Beta-lactamase	Gene	Antibiotic Resistance	No. of isolates with N-fold <1		No. of isolates with N-fold ranging from 1 to 1.99		No. of isolates with N-fold ≥2		Mean fold increase in gene expression in beta-lactamase isolates		p- value	OR (95% CI) (lower-upper)
			^a^R N (%)	^b^S N (%)	^a^R N (%)	^b^S N (%)	^a^R N (%)	^b^S N (%)	^a^R	^b^S		
ESBL	TEM	Cefotaxime	1 (3.33)	9 (30)	2 (6.66)	6 (20)	12 (40)	0	6.89 ± 5.42	1.04 ± 0.36	<0.001*	5.85 (2.97–8.72)
	SHV		0	8 (26.6)	3 (10)	7 (23.3)	12 (40)	0	6.31 ± 4.65	1.06 ± 0.39	<0.001*	5.25 (2.78–7.71)
	CTX-M		0	8 (26.6)	4 (13.3)	7 (23.3)	11 (36.6)	0	4.94 ± 4.08	1.09 ± 0.34	<0.001*	3.85 (1.68–6.01)
	OXA		0	10 (33.3)	4 (13.3)	5 (16.6)	11 (36.6)	0	4.47 ± 4.02	1.00 ± 0.38	<0.001*	3.47 (1.33–5.60)
MBL	NDM-1	Imipenem	1 (3.33)	10 (33.3)	3 (10)	5 (16.6)	11 (36.6)	0	5.09 ± 3.83	0.81 ± 0.39	<0.001*	4.28 (2.24–6.31)
	IMP		0	7 (23.3)	4 (13.3)	8 (26.6)	11 (36.6)	0	2.96 ± 2.57	0.95 ± 0.40	<0.001*	2.01 (0.63–3.39)
	VIM		0	7 (23.3)	5 (16.6)	8 (26.6)	10 (33.3)	0	3.68 ± 2.89	0.87 ± 0.43	<0.001*	2.81 (1.26–4.35)
ABL	ACT	Cefoxitin	0	9 (30)	4 (13.3)	6 (20)	11 (36.6)	0	3.05 ± 1.65	0.95 ± 0.52	<0.001*	2.1 (1.18–3.014)
	CMY		1 (3.33)	10 (33.3)	3 (10)	5 (16.6)	11 (36.6)	0	3.66 ± 3.01	0.87 ± 0.53	<0.001*	2.79 (1.17–4.41)
	DHA^#^		1 (3.33)	19 (63.3)	2 (6.66)	5 (16.6)	3 (10)	0	6.14 ± 5.71	0.89 ± 0.41	<0.001*	5.25 (2.26–8.23)

The mean fold change in relative expression in both resistant and sensitive isolates is also shown.

^a^Resistant; ^b^Sensitive; ^*^significant *p*-value; ^#^only 6 resistant isolates were present.

### Data analysis

**Figure 3:**
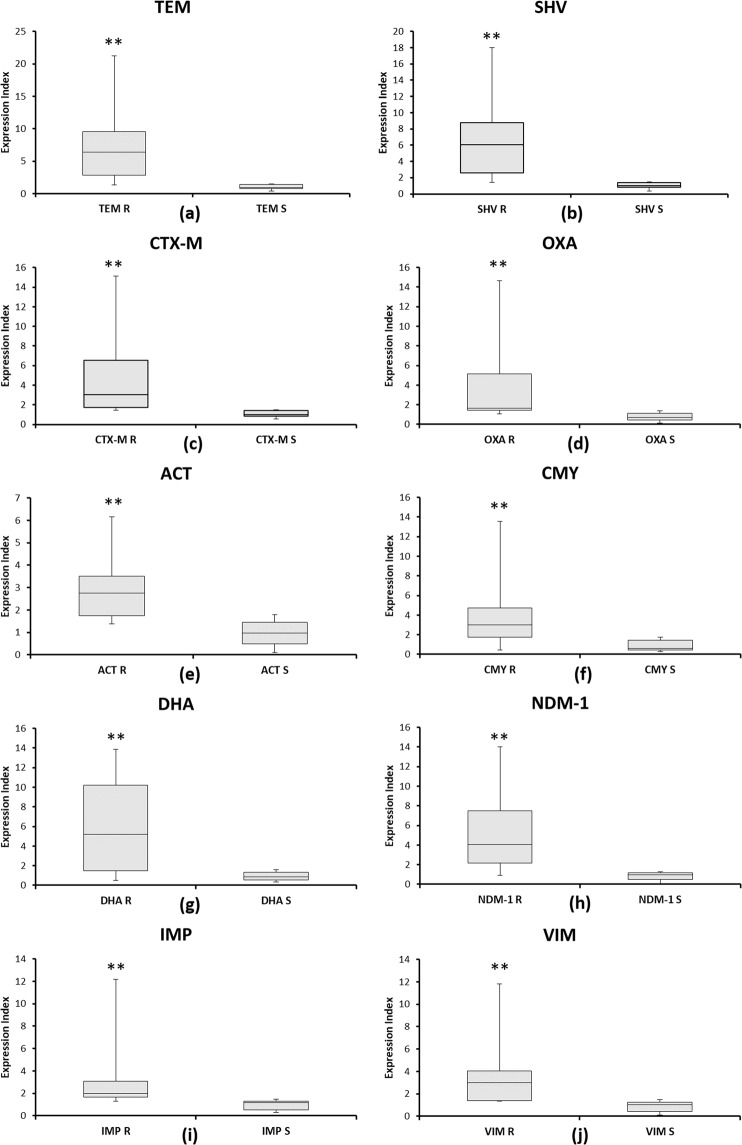
Box plots for β-lactamase (ESBL, MBL and ABL) genes for drug-resistant (R) and sensitive (S) DEC isolates analyzed by the 2^−ddCt^ method. The relative expression levels of (**a**) *TEM*, (**b**) *SHV*, (**c**) *CTX-M*, (**d**) *OXA*, (**e**) *ACT*, (**f**) *CMY*, (**g**) *DHA*, (**h**) *NDM*-1, (**i**) *IMP*, (**j)** *VIM* genes for 15 DEC (each of resistant and sensitive) isolates is shown. The significance levels were derived by Student’s unpaired t-test. Error bars indicate standard deviation. **Indicates *p*-value < 0.001 for all the genes.

Significant differences were observed in the mean expression levels of the β-lactamases genes in resistant and sensitive DEC isolates by the 2^−ddCt^ method as shown in Fig. 3(a–j). The mean and standard error of the mean for all the genes were calculated. For *TEM* gene, resistant isolates presented a 6.89 ± 5.42 fold increase in the expression levels when compared to the sensitive group with 1.04 ± 0.365 fold expression (95% CI: 2.97 to 8.72, p < 0.001); similarly for *SHV* gene, a 6.31 ± 4.65 fold increase compared to 1.06 ± 0.387 (95% CI: 2.78 to 7.72, p < 0.001); for *CTX-M* gene, a 4.94 ± 4.08 fold increase compared to 1.09 ± 0.34 (95% CI: 1.68 to 6.01, p < 0.001); for *OXA* gene, a 4.47 ± 4.018 fold increase compared to 1.00 ± 0.38 (95% CI: 1.33 to 5.60, p < 0.001); for *ACT* gene, a 3.05 ± 1.65 fold increase compared to 0.95 ± 0.52 (95% CI: 1.18 to 3.01, p < 0.001); for *CMY* gene, a 3.66 ± 3.01 fold increase compared to 0.87 ± 0.53 (95% CI: 1.17 to 4.41, p < 0.001); for *DHA* gene, a 6.14 ± 5.71 fold increase compared to 0.89 ± 0.41 (95% CI: 2.27 to 8.23, p < 0.001); for *NDM*-1 gene, a 5.09 ± 3.83 fold increase compared to 0.81 ± 0.39 (95% CI: 2.25 to 6.31, p < 0.001); for *IMP* gene, a 2.96 ± 2.57 fold increase compared to 0.95 ± 0.40 (95% CI: 0.63 to 3.39, p < 0.001); for *VIM* gene, a 3.68 ± 2.89 fold increase compared to 0.87 ± 0.43 (95% CI: 1.26 to 4.35, p < 0.001). Mean range fold change in β-lactamase gene expression in all the isolates is shown in Supplementary File (Fig. S5).

### Analysis of promoter region

**Figure 4:**
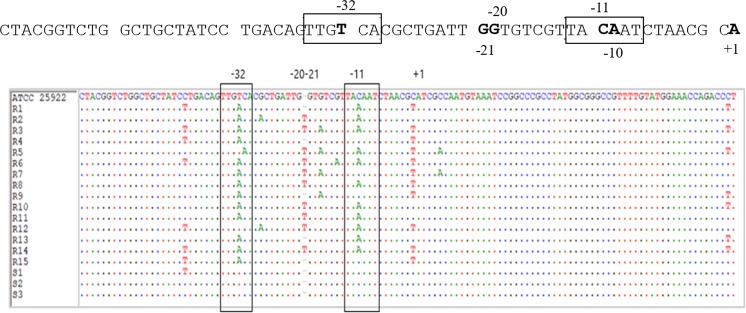
Multiple sequence alignment of 120 bp region of the promoter. Nucleotides and amino acids from the *E. coli* ATCC 25922 (wildtype) sequence that are identical to our test sequences (R = resistant and S = sensitive) are represented by periods. The -35 box and-10 box is underlined (involved in point mutations).

Sequence analysis of the *AmpC* promoter region obtained from the sequencing of PCR amplicons of fifteen resistant isolates showed base substitutions and base insertion (Fig. 4). A transversion of T → A at position −32 changed the wildtype −35 box (TTG**T**CA) to the consensus −35 box sequences (TTG**A**CA), causing overexpression of promoter (10 to 40-folds) when compared to ATCC 25922 (*p* < 0.05); and at position −11 C → T changed the wild-type −10 box from TA**C**AAT to TA**A**AAT, causing 20-fold change in expression level (*p* < 0.05). Point mutations involving transversion of C → T at −42, −1 and +58 positions were also detected causing moderate level of β-lactamase production (1.5 to 8-fold). Apart from that, an insertion of T between −20 and −21 was also observed causing 4 to 6-fold change in gene expression level. Although insignificant, this insertion causes very low level of expression due to its presence in spacer region and it was rare. In sensitive samples, only one out of fifteen showed transversion at −42 position, which is a weak promoter. All these mutations in the *AmpC* coding region are the possible cause of overexpression of mRNA of these target genes in resistant isolates. The sequenced promoter region gene was compared in the GenBank database by BLAST and submitted to the NCBI database (Accession no. MK139014).

## Discussion

*Enterobacteriaceae* have accumulated an extensive array of β-lactamase genes over a period of several years encoding the ESBL, ABL, and more recently carbapenemases. We attempted to analyze, comprehensively, the changing paradigm of antibiotic resistance in infections caused by *E. coli*. Many genes are speculated to be involved in the complicated antibiotic resistance, but the data available is limited. We focused on this aspect to gain an understanding of the genes involved in antibiotic resistance.

The mRNA is synthesized only by viable cells and, therefore, it can be used as a marker of cell viability in bacteria^9,26,27^. Bacterial mRNA is a highly unstable, fragile entity with a very short half-life and needs a harsh working environment and operating conditions. Hence, it is crucial to use the precise amount of mRNA with careful calculations to overcome its loss/compensation to obtain high and consistent yield with high purity^9,28^. Extreme precautions are indispensable for sample preparation to maintain consistent levels of mRNA throughout the process and reduce variability in the results owing to the loss or degradation of the mRNA during preparation. The mRNAs quantification by RT-qPCR is useful in relative comparison of resistance gene expressions. Internal controls serve to eliminate and normalize inter-sample variation during the isolation and reverse transcription steps involving mRNA. Furthermore, it helps to reduce variations in total transcriptional activity between cells.

The expression pattern of β-lactam genes for resistant and non-resistant isolates was analyzed in 15 samples each. The crucial step in the RT-qPCR assay is the variance in the percentage of transcribed mRNA into cDNA, as the efficiency of extracted mRNA can vary for the same targets^28,29^. In between the resistant isolates, we noticed a difference in the levels of expression of all β-lactamase genes. The mRNA expression of 10 β-lactam genes of ESBL, MBL, and ABL classes were found to be high in resistant isolates, except for few isolates where mRNA expression was either negligible (down-regulated) or comparable to the control strains. In the case of drug-sensitive isolates, the mRNA expression level was mostly found down-regulated, except for few isolates where it was comparable to the control strains, suggesting their non-pathogenic nature. However, down-regulation in sensitive strains could be a result of process of genetic gain or development.

Resistance to β-lactams due to the expression of various genes among DEC isolates has become a widespread phenomenon, and their expansion among other members of *Enterobacteriaceae* is increasing. Consequently, this resistance determinant has been reported to spread across the globe^30^. Earlier studies demonstrated that the sub-inhibitory concentrations of antibiotics interfere with the expression of the genes, colonization, and motility of the cell^31^. Under the antibiotic pressure, we observed significant changes in the expression pattern at transcriptional level for all the target genes, which is in agreement with a previous study for *NDM*-1 gene^32^.

The high expression of β-lactamase genes (approximately12 folds) in resistant isolates, as compared to sensitive isolates, indicates their probable role in disease pathogenesis. Their down regulation on the other hand points towards their redundancy. This huge variance in gene expression between the resistant and sensitive isolates, from children up to five years of age, with no regular pattern of expression points out the presence of heterogeneity among the individual isolates. This heterogeneity may play a vital role in disease progression and severity, and survival of the resistant pool. Our observations emphasize the threat of development of β-lactamase mediated resistance in DEC in early childhood.

The role of β-lactamase(s) gene expression has not, noticeably, been correlated with antibiotic resistance; their expression patterns under antibiotic stress can help decipher the possible treatment options especially for patients who suffer from infections due to multiple β-lactamases harboring bacteria. The expression of these genes directly interferes with the treatment and clinical effectiveness of the prescribed drugs. Earlier studies have strongly correlated the copy number with β-lactamase gene expression^33^. Our data support the higher level of transcription of these genes in resistant isolates, and also the potential to predict the response of DEC under antibiotic exposure in real-time. This association of antibiotic resistance and transcriptional expression of the β-lactamase gene(s), as revealed in *E. coli*, can be of immense interest in generating future strategies for downregulating their expression^34^. Whether the bacteria are able to maintain the high expression of these resistance genes on withdrawal of antibiotic pressure needs to be ascertained.

As previously published, constitutive expression of *AmpC* occurs at a lower level in *E. coli*^35^, but various mutations can result in constitutive overexpression of β-lactamases^15,16,36–38^. Our study demonstrated that mutation at position −32 increased *AmpC* expression by 5.98 folds based on RT–qPCR assay. Similarly, mutations at positions −11, −20, −21, −42, −1 and +58 cause increased *AmpC* expression in the range of 1.39 to 13.87 folds, which is in agreement with the previously reported studies^37–39^. Hyper-production of *AmpC* leads to increased resistance to cephalosporins as corroborated in our DEC isolates.

## Conclusion

The present study highlights the insurmountable threat of antibiotic resistance in *E. coli* harboring β-lactamases which provide them additional advantage in survival and augment their pathogenicity. We evidenced that the study of transcription of β-lactamase genes provides a better perception of bacterial responses under different antibiotic concentrations. Also, mutations in *AmpC* promoter region leads to increased transcription of β-lactamase genes in resistant isolates from children. The survival of antibiotic resistant microbes in healthy children and in children without diarrhea represent asymptomatic reservoirs of life-threatening bugs with high dispersion potential. Hence, strict antibiotic policy and prevention of misuse of antibiotics have to be implemented to bring down the rising menace of drug resistance in the community.

## Materials and Methods

### Clinical specimens

The study was conducted on children up to five years of age with symptoms of diarrhea and not receiving antibiotics, and admitted children receiving antibiotic therapy (oral or I/V) for 72 hours or more for reasons other than diarrhea, attending out-patient department of a tertiary care hospital (University College of Medical Sciences and Guru Teg Bahadur Hospital, University of Delhi). Healthy children who were not suffering from diarrhea or any other diseases were also enrolled. Each group consisted of 40 children.

### Ethical considerations

All the experimental protocols were approved by the Institutional Ethics Committee – Human Research (IEC-HR) of the University College of Medical Sciences and Guru Teg Bahadur Hospital, New Delhi. The methods were carried out in accordance with the relevant guidelines. Written informed consent was obtained from the parents/local guardians of children before their enrollment in the study.

### Sample collection and processing

Fresh stool samples were collected in clean, leak-proof, well-labeled, sterile and wide-mouthed plastic containers and were transported immediately to the laboratory for culture. Up to five dark pink colonies (lactose fermentation) with the typical appearance of *E. coli* on MacConkey agar were selected and subjected to conventional biochemical tests for identifying *E. coli* such as gram staining (gram-negative and rod-shaped bacterium), catalase test (+ve), oxidase test (−ve), glucose fermentation with production of gas, fermentation of other sugars (lactose, sucrose, maltose and mannitol), nitrate reduction (+ve; reduces nitrate into nitrite), urease (−ve), Methyl Red Voges Proskauer [MR (+ve) and VP (−ve)], OF glucose test (glucose Fermenter), decarboxylase test [lysine (+ve), arginine (−ve) and ornithine (+ve/−ve)], indole test (+ve), Simon’s citrate (−ve) and hydrogen sulfide (−ve)^40^. PCR for 16SrRNA gene was also performed as an internal quality control for *E. coli*^41^.

### Detection of diarrheagenic *E. coli* (DEC)

All the *E. coli* isolates were checked for the presence of virulence genes of DEC by conventional multiplex PCR method using the following set of genes: *elt* and *est* for ETEC; *eagg* and *east* for EAEC; *eae* for atypical EPEC, and *eae* + *bfp* (*eaf*) for typical EPEC; *stx* and *hyla* for EHEC; *ipah* for EIEC; and *daaE* for DAEC. Primers used for amplifying the sequences were based on previously published literature^25^. The constitutively expressed and highly conserved GAPDH gene in bacteria was used as control in RT-qPCR experiments^42–44^.

### Antibiotic susceptibility testing

Antimicrobial susceptibility testing was performed with 16 antimicrobial agents (HiMedia Laboratories, Mumbai, India) as per CLSI guidelines^45^. The *E. coli* (ATCC) strain 25922 was included as a quality control.

### Identification of β-lactamase producing *E. coli*

The criteria for determination of β-lactamase producing *E. coli* were defined as: the presence of *TEM* (Temoneira), *SHV* (Sulfhydryl variable), *CTX* (Cefotaxime hydrolyzing capabilities), and *OXA* (Oxacillin hydrolyzing capabilities) for ESBL; *NDM-1* (New Delhi Metallo β-lactamase), *IMP* (Imipenem), and *VIM* (Verona integron-encoded Metallo-β-lactamase) for MBL; and *ACT* (*AmpC* type), *CMY* (Cephamycins), and *DHA* (Dhahran Hospital) for ABL class of genes. Primers were selected from previously published literature as shown in Table 2^41,46–50^.

**Table 2:** The DNA sequences of the primers, the size of PCR product and annealing temperature of the genes.

Target Gene	Primer Sequence (5′-3′)	Amplicon Size (bp)	Tm	Reference
**ESBL**				
*TEM*	AGTGCTGCCATAACCATGAGG CTGACTCCCCGTCGTGTAGATA	431	60 °C	**45**
*SHV*	GATGAACGCTTTCCCATGATG CGCTGTTATCGCTCATGGTAA	214		**45**
*OXA*	ATTATCTACAGCAGCGCCAGTG TGCATCCACGTCTTTGGTG	296		**45**
*CTX-M*	GACAAAGAGAGTGCAACGGATG TCAGTGCGATCCAGACGAAA	501		**45**
16 s RNA	CCCCCTGGACGAAGACTGAC ACCGCTGGCAACAAAGGATA	401		**33**
**MBL**				
*NDM-1*	ATTAGCCGCTGCATTGAT CATGTCGAGATAGGAAGTG	154	55 °C	**46**
*IMP*	TTGACACTCCATTTACAG GATTGAGAATTAAGCCACTCT	139		**47**
*VIM*	GATGGTGTTTGGTCGCATA CGAATGCGCAGCACCAG	390		**47**
*GAPDH*	ACTTACGAGCAGATCAAAGC AGTTTCACGAAGTTGTCGTT	170		**48**
**ABL**				
*CMY*	GCTGCTCAAGGAGCACAGGAT CACATTGACATAGGTGTGGTGC	520	60 °C	**49**
*DHA*	AACTTTCACAGGTGTGCTGGGT CCGTACGCATACTGGCTTTGC	405		**49**
*ACT-1*	TCGGTAAAG CCGATGTTG CGG CTT CCA CTG CGG CTG CCA GTT	302		**49**
16 s RNA	CCCCCTGGACGAAGACTGAC ACCGCTGGCAACAAAGGATA	401		**33**

### RNA isolation and cDNA synthesis

Total RNA was isolated from 2 ml of fresh overnight Luria-Bertani (LB) broth culture/peptone water from the stool samples of resistant (resistant to three or more antibiotics), non-resistant and control strains by manual trizol method (Invitrogen, India) according to the manufacturer’s instructions. The RNA was treated with DNase-I (Invitrogen, India) to minimize the risk of DNA contamination. The quantity of extracted RNA was determined by A260 measurements, where reading of 1.0 equals to approximately 40 µg of single-stranded RNA/ml. The purity (A260/A280) of RNA was >1.8 when measured with NanoDrop 2000 spectrophotometer (Thermo Scientific, India). First strand cDNA was prepared from 1 μg of total RNA for all the isolates using 1 μl of oligo (dT) primers, 2.5 μl of 10x cDNA synthesis buffer (Thermo Scientific, India) in a 25 μl of total volume to obtain 40 ng equivalent RNA/μl. The PCR conditions of 25 °C for 10 mins, 50 °C for 50 mins and 85 °C for 5 mins were used. The cDNA was diluted in the ratio of 1:10 using DEPC treated water and stored at −20 °C. The cDNA from *E. coli* isolates from healthy children was used as negative control and was processed along with other resistant and sensitive isolates. The 16SrRNA and GAPDH genes were used as internal controls^51–53^. The intensity was expressed as a value relative to that of the 16SrRNA^54^.

### Quantitative Real time PCR (qPCR)

Fifteen isolates each of the resistant and sensitive *E. coli* were taken up for relative quantification of the β-lactamases genes along with 15 control strains. The qPCR was performed using an SYBR Green qPCR kit (Roche Diagnostics, USA) and a Light Cycler 480-II system (Roche Diagnostics, USA) was used to measure the relative transcript levels of the 10 β-lactamase genes in resistant and non-resistant isolates. Initially, primers were standardized with cDNA using conventional PCR.

A total of 20 μl reaction volume was used including 3 μl cDNA, 10 μl of (2x) Syber green master mix, 1 μl each of forward and reverse primers (10 μM) and nuclease-free water to make up the volume. No-template control (NTC) was run with each reaction for each gene. The NTC were PCR mixtures containing water in place of template cDNA. The genes were amplified with an initial denaturation of 95 °C for 5 mins, followed by 40 cycles of 95 °C for 20 secs., 55 °C or 60 °C for 30 secs., and 72 °C for 20 secs. High number of PCR cycles were run to ensure the saturation of all the reactions and to obtain a sufficient amount of product. The acquisition temperature for fluorescence was 72 °C for all the genes. The RT-qPCR was performed in triplicates to minimize any errors caused by handling and average of all the three values was taken as final.

To validate amplification specificity further, a single cycle of the melting curve was performed with 95 °C for 10 secs. and 65 °C for 20 secs. with the continuous acquisition (from 65 °C gradually increasing by 0.1 °C/sec. to 95 °C, with fluorescence data acquisition every 1 sec.) and finally cooling at 40 °C. The instrument automatically calculates melting curves by converting them into melting peaks by plotting the negative derivative of fluorescence measured at 533 nm and generates melting peaks by plotting about temperature (2dF/dT). The melting curves were calculated for all the isolates using qPCR. Relative gene expression (fold change) was calculated using the formula 2^−ddCt^ ^55^. Constantly expressed genes were used as internal controls in relative quantification studies. Threshold cycle value (Ct value) is defined as the PCR cycle where the fluorescence signal increases above the background threshold^29^.

### Transcriptional assays

The greater the quantity of target cDNA in the starting material, the faster a significant increase in fluorescent signal will appear, yielding a lower Ct^56^. The Ct values were read and recorded for ESBL, MBL and ABL, and the endogenous genes in resistant, non-resistant and control strains. An average of Ct values for all 15 isolates in the control strains was used for normalization. The difference between Ct of the drug-resistant or drug-sensitive genes and the internal control genes gave the ΔCt values. Subsequently, ΔΔCt for all the resistant and sensitive DEC isolates were calculated, which is the difference between the ΔCt of the resistant or sensitive isolates and the ΔCt of the control strains. Relative quantification or the fold change in expression of the β-lactamase genes was calculated as N-fold which is equal to 2^−ddCt^ for all isolates versus the control strains^55^.

### Sequencing and data submission

Sequencing of the β-lactamase genes (amplified by the primers shown in Table 2) was performed commercially (Helix Biosciences, Bangalore, India). To increase the accuracy of the results, sequencing was performed with both forward and reverse primers and sequences were compared against GenBank database by using BLAST and submitted to the NCBI database.

### DNA sequence analysis of promoter region

DNA was extracted from *E. coli* colonies by using the commercial kit (Real Biotech Corporation, Taiwan) following the manufacturer’s instructions. For *AmpC* promoter mutation analysis, a 271-bp fragment was amplified using a forward primer (5′-GATCGTTCTGCCGCTGTG-3′) and a reverse primer (5′GGGCAGCAAATGTGGAGCAA-3′)^57^. PCR amplicons were sequenced commercially (Helix Biosciences, Bangalore, India). All isolates were characterized genetically by mutational analysis. The promoter sequences were compared to the wild-type sequence from *E. coli* (ATCC 25922), used as a promoter control by multiple sequence alignment using the BioEdit online tool (version 7.0.5).

### Statistical analysis

Statistical analysis was done using SigmaStat statistical software package (SPSS). Student’s unpaired ‘t’ test was performed to analyze the data derived by the 2^−ddCt^ method. *p-*value of less than 0.05 was considered as significant. The transcript levels of the target genes for both resistant and sensitive isolates were presented as mean ± SEM (standard error of the mean).

## Supplementary information

Supplementary Information file
