# Supplementary material for: Transcriptome analysis of beta-lactamase genes in diarrheagenic Escherichia coli
Source: Sci Rep. 2019 Mar 6;9:3626. doi: 10.1038/s41598-019-40279-1 (PMC6403342; doi:10.1038/s41598-019-40279-1)

Transcriptome analysis of β-lactamase genes in diarrheagenic *Escherichia coli*

Authors: *Taru Singh, Praveen Kumar Singh, Shukla Das, Sayim Wani, Arshad Jawed, Sajad Ahmad Dar*

Table S1: Frequency of resistance to antimicrobial agents of *E. coli* isolates from three study groups.

Antibiotics	Group 1 n=40 (%)	Group 2 n=40 (%)	Group 3 n=40 (%)	Total n =120 (%)	*p*-value
Norfloxacin (10µg)	9(22.5)	7(17.5)	10(25)	26(21.66)	0.709
Cefotaxime (30µg)	27(67.5)	33(82.5)	7((17.5)	67(55.83)	0.00*
Imipenem (10µg)	12(32.5)	5(12.5)	1(2.5)	18 (15)	0.002*
Meropenem (10µg)	2(5)	1(2.5)	0	3(2.5)	0.358
Ceftazidime (30µg)	8(20)	4(10)	0	12(10)	0.011*
Azetronam (30µg)	5(12.5)	6(15)	0	11(9.1)	0.044*
Nalidixic acid (30µg)	8(20)	0	0	14(11.66)	0.00*
Amoxicillin + Clavulanic acid (20/10µg)	1(2.5)	2(5)	0	3(2.5)	0.358
Gentamicin (10µg)	15(37.5)	14(35)	2(5)	31(25.83)	0.01*
Ciprofloxacin (5µg)	7(17.5)	4(10)	1(2.5)	12(10)	0.082
Ampicillin (10µg)	17 (42.5)	9 (22.5)	4 (10)	30 (25)	0.01*
Amikacin (30µg)	9(22.5)	14(35)	0	23(19.1)	0.00*
Polymyxin B (300µg)	1(2.5)	0	0	1(0.83)	0.364
Cefotaxime + Clavulanic acid (30/10µg)	0	1(2.5)	0	1(0.83)	0.364
Ceftriaxone (30µg)	0	1(2.5)	1(2.5)	2(1.66)	0.601
Piperacillin + Tazobactam (100/10µg)	10(25)	9(22.5)	2(5)	21(17.5)	0.037*

*Statistically significant; Antibiotic frequencies are presented as absolute numbers (n) with percentage in parentheses

Figure S1: Real-time PCR assay for mRNA expression of four Extended Spectrum β-Lactamase (ESBL) genes: (a) Amplified peaks for TEM, SHV, CTX-M and OXA genes in cefotaxime resistant isolates; X-axis representing melting temperature and the Y-axis representing the rate of change of fluorescence over time; **(b)** X-axis representing number of cycles and the Y-axis representing the rate of change of fluorescence over time.

(a)

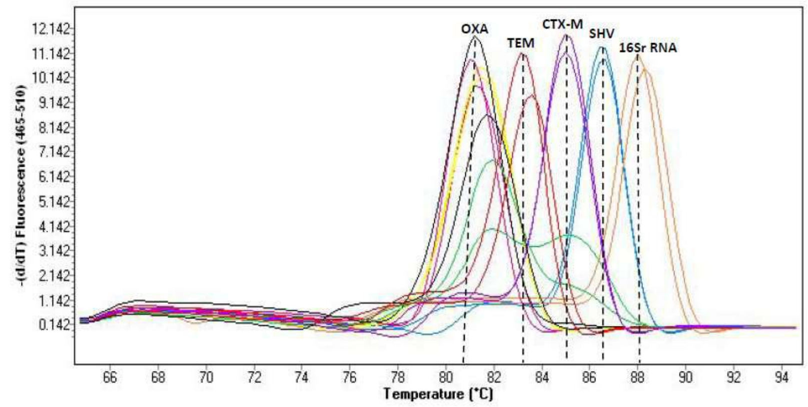

(b)

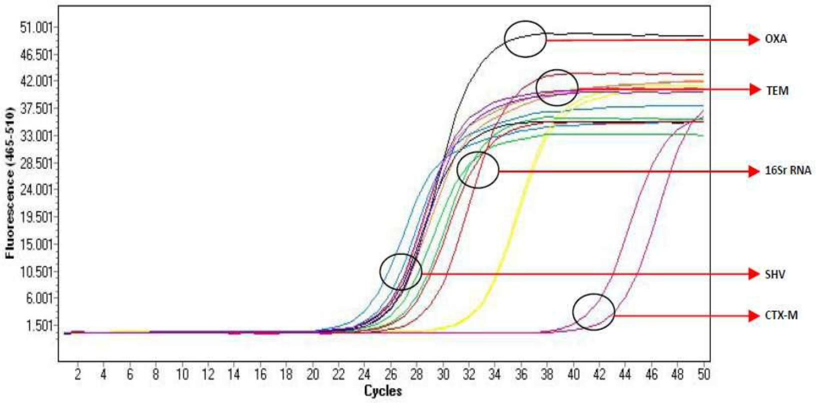

Figure S2: Real-time PCR assay for mRNA expression of three Metallo β-Lactamase (MBL) genes: (a) Amplified peaks for NDM, IMP and VIM genes in meropenem resistant isolates; X-axis representing melting temperature and the Y-axis representing the rate of change of fluorescence over time; **(b)** X-axis representing number of cycles and the Y-axis representing the rate of change of fluorescence over time.

(a)

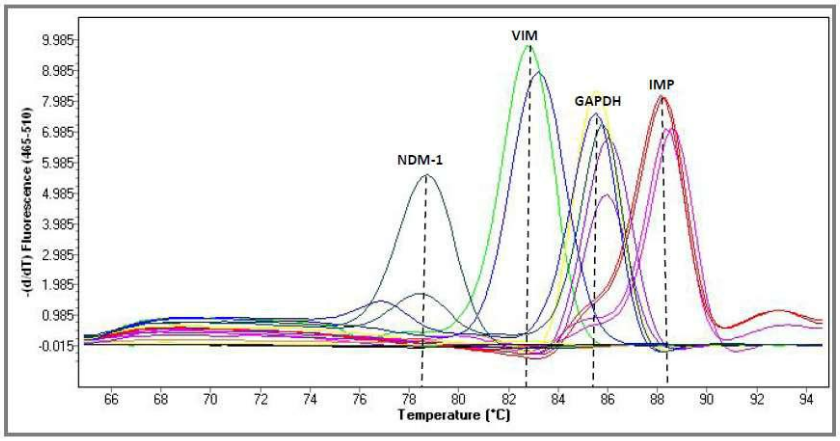

(b)

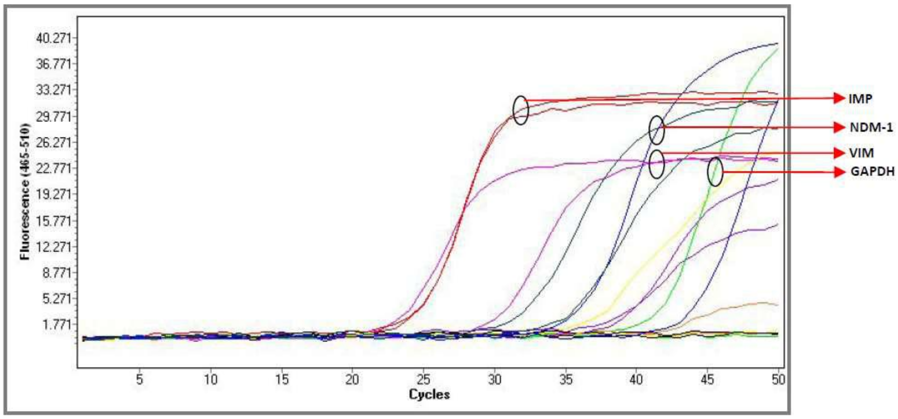

Figure S3: Real-time PCR assay for mRNA expression of three AmpC β-Lactamase (ABL) genes: (a) Amplified peaks for ACT, CMY and DHA genes in cefoxitin resistant isolates; X-axis representing melting temperature and the Y-axis representing the rate of change of fluorescence over time; **(b)** X-axis representing number of cycles and the Y-axis representing the rate of change of fluorescence over time.

(a)

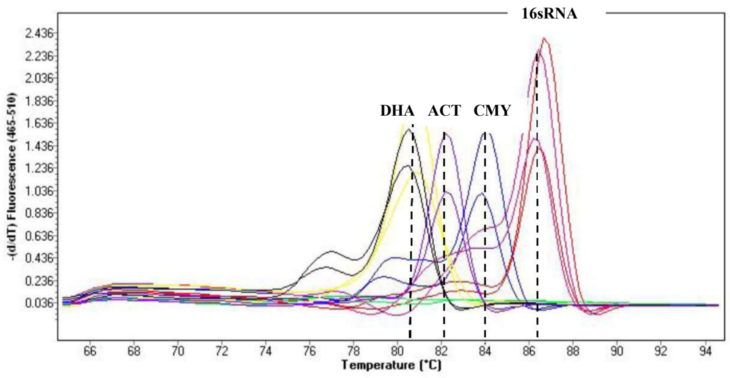

(b)

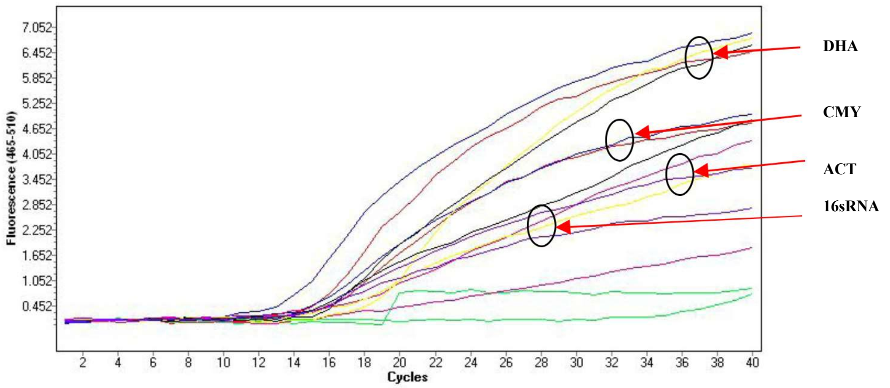

Figure S4: The mean fold change in relative expression of each β-lactamase drug-resistant **(a)** and sensitive **(b)** genes, in comparison to control group isolates. Error bars indicate standard deviation. **(c)** Comparative mean fold change in expression of β-lactamase genes in resistant and sensitive isolates. Error bars indicate standard error.

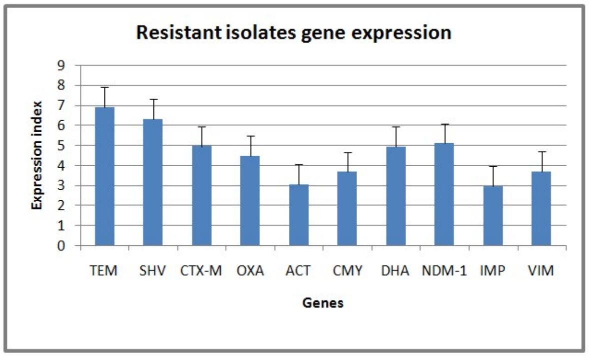

(a)

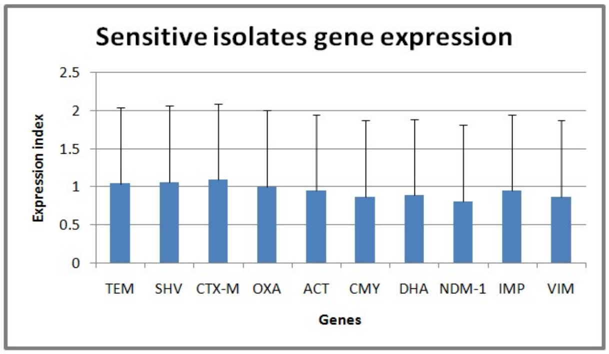

(b)

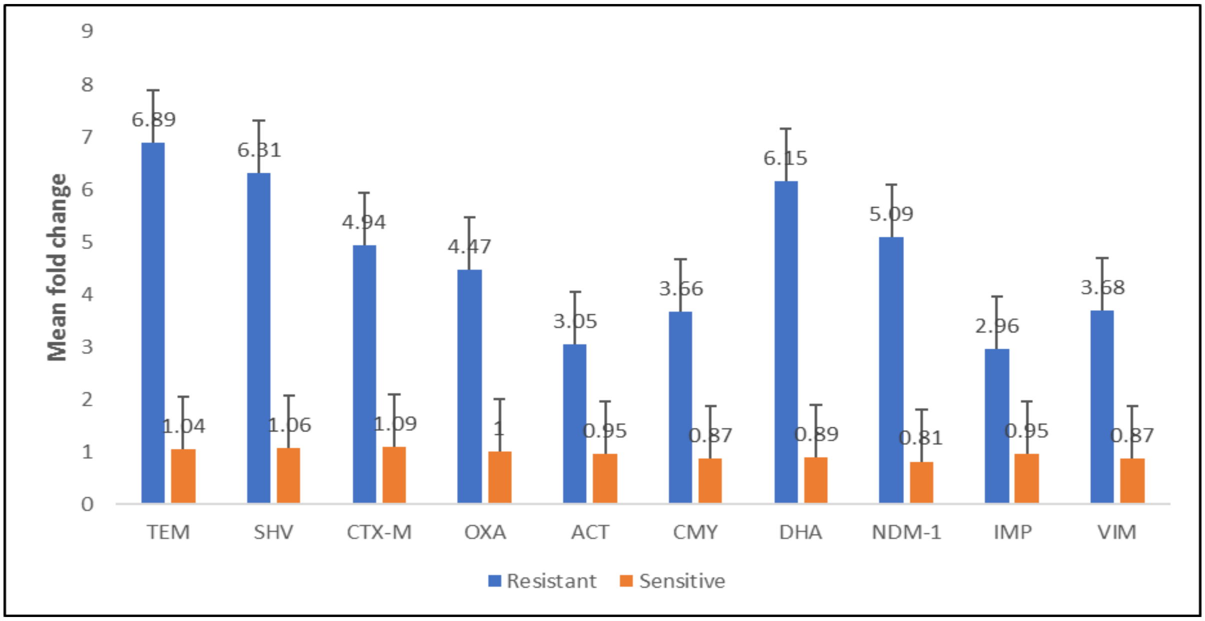

(c)

Figure S5: Forest plot for β-lactamase genes showing range fold change in all the isolates.

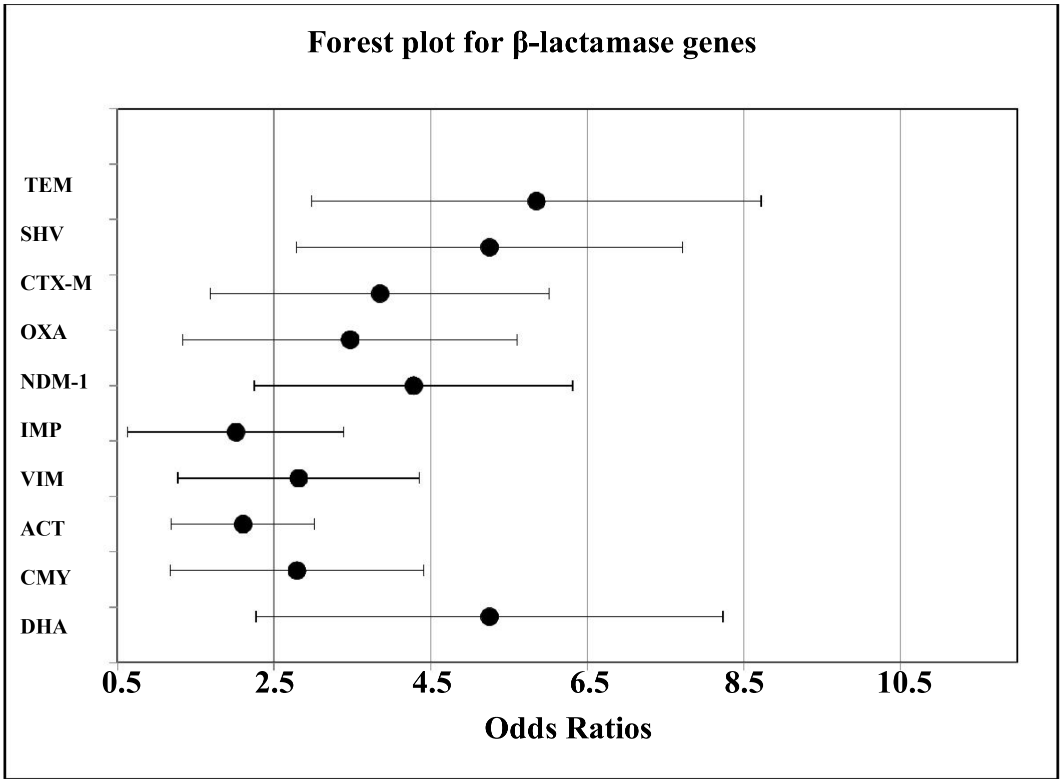

Supplement: Supplementary file 1 — Supplementary Information file [file 41598_2019_40279_MOESM1_ESM.pdf]
